# Relationship Between Cortical Excitability Changes and Cortical Thickness in Subcortical Chronic Stroke

**DOI:** 10.3389/fneur.2022.802113

**Published:** 2022-03-08

**Authors:** José A. Graterol Pérez, Stephanie Guder, Chi-un Choe, Christian Gerloff, Robert Schulz

**Affiliations:** Department of Neurology, University Medical Center Hamburg-Eppendorf, Hamburg, Germany

**Keywords:** gray matter, cortex, recovery, motor, MRI, cortical excitability

## Abstract

Ischemic stroke leads to excitability changes of the motor network as probed by means of transcranial magnetic stimulation (TMS). There is still limited data that shows to what extent structural alterations of the motor network might be linked to excitability changes. Previous results argue that the microstructural state of specific corticofugal motor tracts such as the corticospinal tract associate with cortical excitability in chronic stroke patients. The relationship between changes of cortical anatomy after stroke, as operationalized by means of decreases or increases in local cortical thickness (CT), has scarcely been addressed. In the present study, we re-analyzed TMS data and recruitment curve properties of motor evoked potentials and CT data in a group of 14 well-recovered chronic stroke patients with isolated supratentorial subcortical lesions. CT data of the stroke patients were compared to CT data of 17 healthy controls. Whole-brain and region-of-interest based analyses were conducted to relate CT data to measures of motor cortical excitability and clinical data. We found that stroke patients exhibited significantly reduced CT not only in the ipsilesional primary motor cortex but also in numerous secondary motor and non-motor brain regions, particularly in the ipsilesional hemisphere including areas along the central sulcus, the inferior frontal sulcus, the intraparietal sulcus, and cingulate cortices. We could not detect any significant relationship between the extent of CT reduction and stroke-related excitability changes of the motor network or clinical scores.

## Introduction

Ischemic stroke leads to time- and recovery-dependent changes of motor cortical excitability which can be probed by means of transcranial magnetic stimulation (TMS). Motor evoked potentials (MEP) and recruitment curve properties have been considered as surrogates for the functional state of the motor network, including important corticofugal motor pathways such as the corticospinal tract (CST). Studies have related excitability measures to motor deficits and recovery processes after stroke (1–7). Over time, the field has moved from unimodal approaches to multimodal analyses of brain structure and function to better understand intersubject variability in stroke recovery (8). For instance, one study combined TMS and MRI to assess cortical excitability, interregional connectivity and damage to the CST and found that these factors accounted for more than 80% of the variance in functional impairment in chronic stroke patients (9). Other studies have recently evidenced significant relationships between CST microstructure and cortical excitability (10, 11).

Apart from important motor pathways, such as the CST, stroke-related alterations of brain structure also affect local cortical anatomy: For instance, cortical thinning has been observed in primary and secondary motor and non-motor brain regions of the ipsi- and contralesional hemispheres (5, 12–18), predominantly in cortices directly connected to the stroke lesion (14, 19) or in the deepest layers of the motor cortices (13). By showing gradual cortical thickening in frontal and temporal cortices (20) or increases of cortical gray matter volume (21, 22), other studies have argued for the existence of neuroplastic brain alterations after stroke to promote recovery processes. However, the precise relationship between cortical thickness (CT) alterations—either loss or gain in CT—and motor recovery remains under debate. There are studies which have found significant associations between cortical anatomy and clinical scores (13, 16, 21, 22) and others which have not (5, 14, 15, 19, 20).

Given these data, the question arises whether CT alterations, particularly located in key motor areas, might explain intersubject variability in excitability of the motor network. Specifically, these areas might comprise the primary motor cortex and also frontal and parietal cortices of the ipsilesional hemisphere since studies have repeatedly shown that the parietofrontal motor network might be critically involved in motor functioning and recovery processes after stroke (23–25). The regional analysis of CT could add information at the cortex level to answer this question, thus supporting available data from previous functional imaging studies or structural imaging studies which focused on corticofugal or interregional corticocortical motor tracts (8). In fact, only a few studies have explored potential associations between CT and TMS-based measures of cortical excitability in stroke patients. For hand dexterity, Borich et al. found that the CT of the precentral gyrus together with cortical excitability were informative predictors. However, the authors did not address any potential interrelationship between both variables (26). Another study compared CT data of the ipsilesional primary motor cortex with its excitability but did not detect any association. As potential limitations, this analysis was constrained to the primary motor cortex and included patients with subcortical strokes but also patients with direct cortex involvement. CT was thinner in patients with cortical strokes (5). Notably, inter-study variability in the composition of the patient groups with lesion locations with (5, 16, 27, 28) or without direct damage to the cortex (12–15, 17–19, 29, 30) might be a relevant influential factor for variability in structure-function relationships. A comparison of different methods to estimate CT has indicated that directly damaged cortical areas exhibit an increased risk for invalid CT values due to local effects of the stroke lesion (31).

The present study was designed to shed new light on the potential relationship between regional CT alterations, changes of cortical excitability, and residual motor function after stroke. For that, we reanalyzed available TMS, MRI, and clinical data of a group of supratentorial subcortical chronic stroke patients. Specifically, high-resolution structural MRI data was used to quantify CT of the ipsilesional and contralesional hemispheres. Data of healthy participants of similar age and gender were used to determine brain regions with significant loss or gain in CT in the stroke cohort. Whole-brain and region-of-interest (ROI) based analyses were conducted to relate CT data to measures of cortical excitability and clinical data.

## Methods

### Participants and Clinical Testing

This study is based on the cohort of chronic stroke patients of our previous report on the influence of cortico-cerebellar structural connectivity on excitability of the motor network (10). The original inclusion criteria were: first-ever supratentorial ischemic stroke with persistent hand motor impairment in the chronic stage of recovery (≥ 6 months), 18 years of age or older, and no contraindication for TMS or MRI. In total, 18 patients were included in that study. In the present work, four patients were excluded due to cortical lesions that could have affected the interpretation of the results. Seventeen healthy participants of similar age and gender served as the control group. Distribution of stroke hemispheres regarding the dominant and non-dominant hemispheres were taken into account. Fourteen control participants were treated as having their dominant hemisphere being pseudo-affected by the stroke. All participants gave written informed consent according to the Declaration of Helsinki to participate in the study which was approved by the local ethics committee (PV5357). Patients underwent clinical testing including grip and pinch forces, Fugl-Meyer assessment of the upper extremity (UEFM), and the nine-hole peg test (NHP). Grip and pinch forces and NHP performances were obtained from both the affected and unaffected hands. These data have been already introduced by our previous report and are taken 1:1 (10).

### TMS Data Acquisition and Analysis

Details of the TMS methodology can be found in our original publication (10). In brief, a Magstim 200 magnetic stimulator with a 70-mm figure-eight coil and electromyographic electrodes placed over the first dorsal interosseous muscle on both hands in a belly-tendon montage was used to collect MEP. The resting motor threshold (RMT) at the hotspot was determined to the nearest 1% of the maximum stimulator output (32). To calculate the properties of the MEP's recruitment curve (RC), blocks of 11 stimuli, in a pseudorandomized order to avoid hysteresis effects and with intensities ranging from 90 to 160% of RMT, were administered. RC data were acquired in the affected (AH) and unaffected hemispheres (UH). Signal software 4.05 (Cambridge, Electronic Design, Cambridge, UK) was used to analyze the data. The first trial of each block was discarded due to the possibility that the initial MEP showed higher amplitudes than subsequent responses. At least 5 trials of every intensity level (90–160% RMT) were used to fit the RC, except for one patient and one control in which a reduced range (90–150%) was used due to a high stimulation intensity at 160%. The RC data were plotted as intensity vs. MEP size (peak-to-peak amplitude). Each individual intensity was then fitted into a sigmoid Boltzmann function which allowed to estimate MEP_max_ (plateau of RC) and Slope_max_ (maximum slope of RC), separately for AH and UH as stroke-related alterations has been evidenced for these two measures. Importantly, these data are taken 1:1 from our previous report which should be consulted by the interest reader for further details (10).

### Brain Imaging and Cortical Thickness Analyses

A 3T Siemens Skyra MRI scanner (Siemens, Erlangen, Germany) and a 32-channel head coil were used to acquire high-resolution T1-weighted anatomical images by means of a three-dimensional magnetization-prepared, rapid acquisition gradient-echo sequence with the following parameters: TR = 2,500 ms, TE = 2.12 ms, FOV = 240 mm, 256 coronal slices with a voxel size of 0.8 x 0.8 x 0.9 mm. Datasets were processed with Freesurfer version 6.0.0 (http://surfer.nmr.mgh.harvard.edu/) using the default options to measure CT (33, 34). The reconstructions were visually inspected and, if required, manually corrected following established recommendations from the Freesurfer's documentation. To compare affected (AH) and unaffected hemispheres (UH), all images were registered to the Freesurfer's common space symmetrical template, *fsaverage_sym*, that allowed to flip all lesions to the left hemisphere (35). Therefore, in this study we treated the left hemisphere as the AH and the right hemisphere as the UH. Surface data were smoothed with a full-width-half-maximum Gaussian kernel of 10 mm.

### Statistics

For group comparisons and correlative analyses with clinical and TMS data, we first followed a whole-brain approach. Using the Freesurfer's *mri_glmfit* utility, general linear models are specified for the whole brain's surface, which comprises CT estimates for every vertex of every subject. To evaluate CT differences between patients and controls, a first model with CT as the dependent variable was fit treating group as the factor of interest. Color-coded statistical parametric maps were displayed using *freeview*. To assess the relationship between CT and cortical excitability or motor function, similar separate models were fit with TMS measures of AH or clinical data of the affected hand treated as the independent variables. All models were adjusted for the effect of age. *P*-values in every model were corrected for multiple comparisons using a Z Monte Carlo simulation over 10,000 iterations with a cluster-forming threshold of *P* < 0.0001 (36). Statistical significance was assumed at cluster-wise corrected *P*-values < 0.05. Size of clusters and number of significant vertices are given in the results section.

In addition to these surface-based whole-brain analyses, a ROI analysis of primary and secondary motor areas was also conducted using R statistical package (version 4.0.3, https://www.r-project.org/). Mean CT values were extracted from clusters of the parietofrontal motor network and cingulate cortices exhibiting a significant loss in CT in the stroke patients compared to healthy controls. These data (now as independent explanatory variable of interest) were fit into separate multiple linear regression models to explain variability in cortical excitability (Slope_max_, MEP_max_) and clinical scores (maximum grip and pinch force, NHP of the affected hands, UEFM). The respective data of the unaffected hemisphere or hand and age were treated as covariates in line with our previous report (10). Slope_max_ and MEP_max_ values were log-transformed to improve data distribution. Models were checked for normal distribution of residuals and relevant multi-collinearity was excluded by estimating the variation inflation factor. Cook's distance was used to check for outliers. Statistical significance was determined as *P* < 0.05. *P*-values are presented as uncorrected values.

## Results

### Demographic and Clinical Data

**Figure 1:**
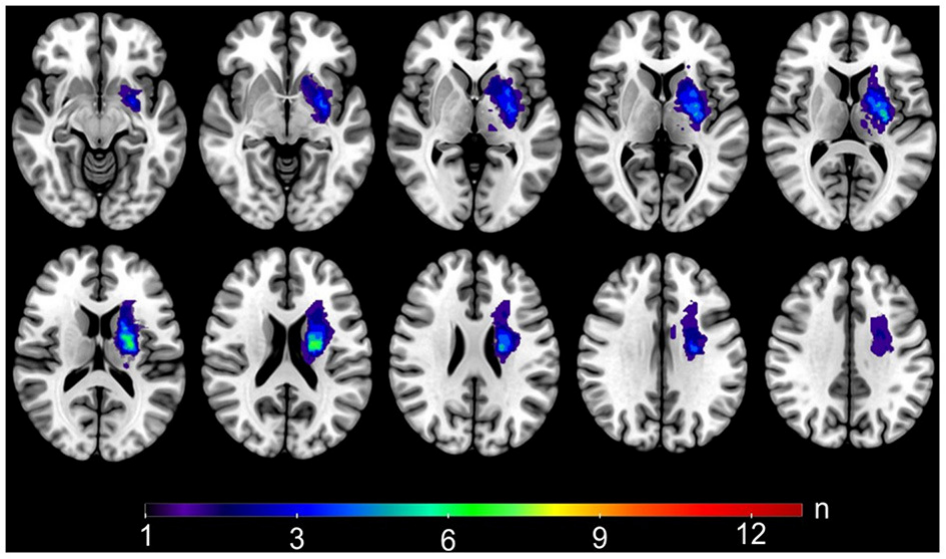
Stroke lesions. Lesion mask overlap showing the distribution of the stroke lesions on a standard MNI template. For visualization purposes, all the masks were registered to the MNI space and flipped to the left hemisphere. Warm colors show areas of bigger overlap.

Analyses were conducted in 14 patients (12 males, mean age 66.86 years, mean 41 months after stroke, one left-handed, 12 patients with lesions on their dominant hemisphere, 6 lacunar strokes). Demographic and clinical data of the patients and controls are given in Table 1. The distribution of the stroke lesions is shown in Figure 1.

**Table 1:** Demographic and clinical data.

**ID**	**Age**	**Gender**	**Tas (months)**	**DoHe**	**AH**	**Stroke** **location**	**LAC**	**Grip (Kg)**		**Pinch (Kg)**		**NHP (pegs/s)**		**UEFM**
								**AH**	**UH**	**AH**	**UH**	**AH**	**UH**	
1	78	F	20	L	L	BG, IC	No	19.33	24.00	7.00	7.50	0.77	0.78	66
2	74	M	20	L	L	CR	Yes	42.00	34.67	10.67	8.83	0.57	0.54	66
3	55	M	66	L	L	BG, IC	No	46.33	45.33	12.17	11.33	0.65	0.89	66
4	75	M	58	L	R	PLIC	No	26.67	40.67	7.50	12.00	0.33	0.84	39
5	61	M	75	L	L	BG, IC	No	31.00	42.67	8.00	9.83	0.51	0.85	47
6	76	M	80	L	L	BG, IC	No	24.33	31.67	8.17	8.50	0.44	0.78	50
7	61	M	88	L	L	PLIC	No	31.00	34.67	8.33	9.67	0.65	0.67	64
8	73	M	62	R	R	BG, IC	No	31.33	35.33	9.83	10.67	0.60	0.72	63
9	60	M	9	L	L	TC	Yes	35.33	43.33	9.00	8.17	0.59	0.75	55
10	58	M	31	L	L	TC	Yes	24.00	22.33	7.17	6.17	0.91	0.86	66
11	64	M	7	L	R	BG, IC	Yes	4.33	21.33	4.00	6.67	0.50	0.79	52
12	63	M	11	L	L	BG, CR	No	20.33	46.00	3.00	8.00	0.53	1.00	51
13	84	F	23	L	L	BG, CR	Yes	9.33	15.00	4.00	6.33	0.43	0.73	39
14	54	M	29	L	L	CR, PLIC	Yes	17.33	42.00	6.33	8.83	0.57	0.73	59
Mean Stroke	66.86	M:86%	41.36	L:13	L:11	—	LAC:6	25.90^*^	34.21	7.51	8.75	0.58^**^	0.78	55.93
SD Stroke	±9.53	—	±28.78	—	—	—	—	±11.57	±10.03	±2.59	±1.80	±0.15	±0.11	±9.84
Mean Control	66.76	M:82%	—	L:16	L:13	—	—	34.80	33.22	7.33	7.18	0.77	0.73	66.00^†^
SD Control	±8.04	—	—	—	—	—	—	±12.17	±10.79	±2.42	±2.57	±0.18	±0.13	±0.00

*Age in years, gender (M, male; F, female), time after stroke (tas, in months), dominant (DoHe) and affected (AH) hemisphere, stroke location (BG, basal ganglia; CR, Corona radiate; IC, internal capsule; TC, thalamocapsular; PLIC, posterior limb of the internal capsile), lacunar infarct (LAC), absolute grip and pinch force values for the affected (AH) and the unaffected (UH) hemisphere, nine-hole peg test (NHP), and the Fugl-Meyer assessment of the upper extremity. Significant group differences are indicated by asterisks*

(* *p ≤ 0.05*

** *p ≤ 0.01)*.

† *UEFM score of 66 was assumed for controls*.

### Cortical Thickness in Stroke Patients

**Figure 2:**
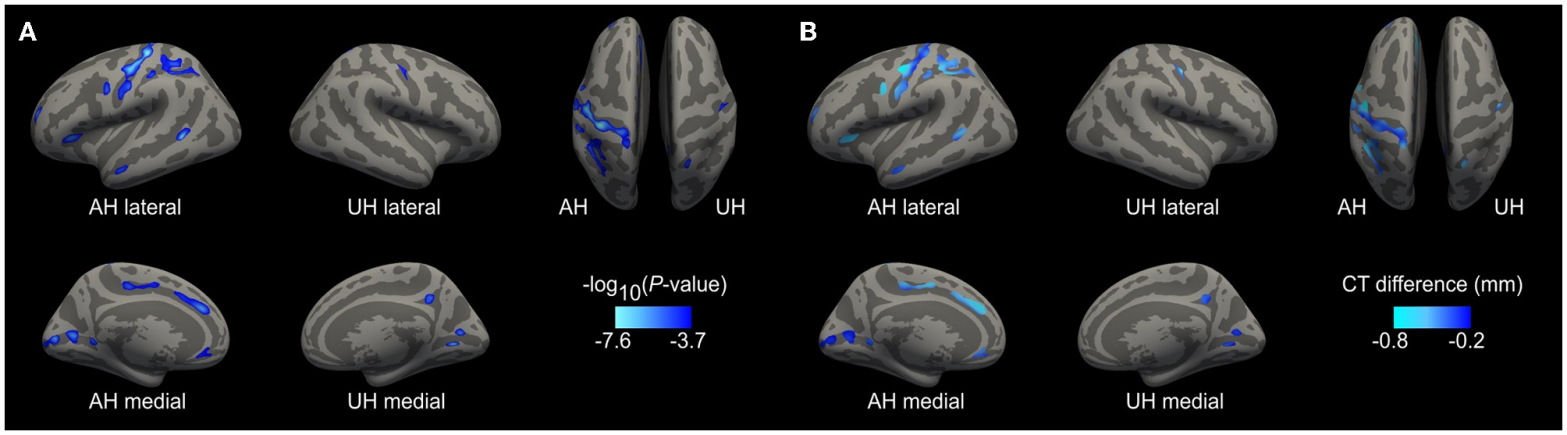
Cortical thickness reductions after stroke. Whole-brain analysis results from comparison of patients with controls. For visualization purposes, the left hemisphere is modeled as the affected hemisphere (AH) and the right hemisphere as the unaffected hemisphere (UH). **(A)** Show clusters of significant CT thinning in the patients. The scale displays values of -log_10_ (*P*-value). Lighter blue tones mean higher significance. **(B)** Display the vertex-wise difference of CT values in mm (patients-controls) using the significant clusters as masks. Lighter blue shows areas of increased cortical thinning.

Whole-brain analysis revealed a significantly reduced CT in patients when compared to controls, after accounting for age, in multiple cortical areas with predominance of the ipsilesional hemisphere (Figure 2). The largest clusters of significant CT reductions were found in the ipsilesional pre-, post-, and paracentral gyri, in the central and paracentral sulci, the cuneal gyrus and the calcarine and parieto-occipital sulci as well as in the superior parietal gyrus and postcentral sulcus. In the contralesional hemisphere, the extent of CT reduction was rather moderate and did only reach statistical significance in the central sulcus, the cuneal gyrus, and in the calcarine sulcus. Tables 2, 3 summarize cluster locations and sizes, cluster-related averaged CT values for patients and controls, and absolute mean group differences. There were no significant CT increases in patients compared to controls in the present cohort.

**Table 2:** Location of the significant CT clusters in the AH of patients when compared to controls.

**Number**	**Location**	**Size (mm^2^)**	**no. of vertices**	**Mean CT Patients (SD)**	**Mean CT Controls (SD)**	**Mean CT Difference**
1^*^^#^	Paracentral gyrus Central and paracentral sulci	1370.89	3,469	1.55 (0.21)	1.99 (0.31)	−0.44
2^*^	Superior parietal gyrus Postcentral, and intraparietal sulci	825.83	1,868	1.91 (0.19)	2.39 (0.24)	−0.48
3^*^	Superior frontal, anterior cingulate, and middle anterior cingulate gyri Anterior cingulate and middle anterior cingulate sulci	355.55	558	2.47 (0.16)	3.01 (0.18)	−0.54
4	Cuneal gyrus Calcarine sulcus	323.06	504	1.52 (0.10)	1.81 (0.11)	−0.29
5	Calcarine sulcus	277.62	372	1.53 (0.08)	1.81 (0.09)	−0.28
6^*^	Middle posterior cingulate gyrus Middle posterior cingulate and marginal cingulate sulci	228.57	541	2.22 (0.17)	2.71 (0.22)	−0.49
7	Lateral fissure and superior circular sulcus	185.07	407	2.07 (0.14)	2.64 (0.13)	−0.57
8	Middle temporal gyrus Superior temporal sulcus	162.32	253	2.51 (0.13)	2.98 (0.16)	−0.47
9	Middle frontal sulcus	156.42	221	2.01 (0.08)	2.43 (0.11)	−0.42
10	Calcarine sulcus	112.66	242	2.09 (0.25)	2.46 (0.27)	−0.37
11	Anterior cingulate gyrus and sulcus	112.56	160	2.32 (0.13)	2.74 (0.17)	−0.42
12^*^	Precentral gyrus Inferior part of the precentral sulcus	109.58	231	2.33 (0.16)	2.93 (0.18)	−0.6
13^*^	Postcentral sulcus	102.7	271	2 (0.10)	2.45 (0.14)	−0.45
14	Middle temporal gyrus Superior temporal sulcus	98.59	152	2.68 (0.14)	3.06 (0.14)	−0.38

*All clusters are corrected for multiple comparisons (cluster-wise corrected P-values <0.05)*.

* *Clusters were selected for ROI analysis*.

# *This large cluster in the surface analysis has been divided into Clusters 1 and 2 for the ROI analysis*.

**Table 3:** Location of the significant CT clusters in the UH of patients when compared to controls.

**Number**	**Location**	**Size (mm^2^)**	**no. of vertices**	**Mean CT Patients (SD)**	**Mean CT Controls (SD)**	**Mean CT Difference**
1	Cuneal gyrus Calcarine sulcus	148.75	180	1.49 (0.08)	1.8 (0.09)	−0.31
2	Subparietal sulcus	126.12	267	2.31 (0.11)	2.69 (0.1)	−0.38
3	Medial lingual gyrus Calcarine gyrus	125.8	188	1.45 (0.07)	1.79 (0.09)	−0.34
4^*^	Superior parietal gyrus	85.01	151	1.98 (0.11)	2.52 (0.15)	−0.54
5^*^	Central sulcus	84.71	221	1.75 (0.15)	2.21 (0.2)	−0.46

*All clusters are corrected for multiple comparisons (cluster-wise corrected P-values < 0.05)*.

* *Clusters were selected for ROI analysis*.

**Figure 3:**
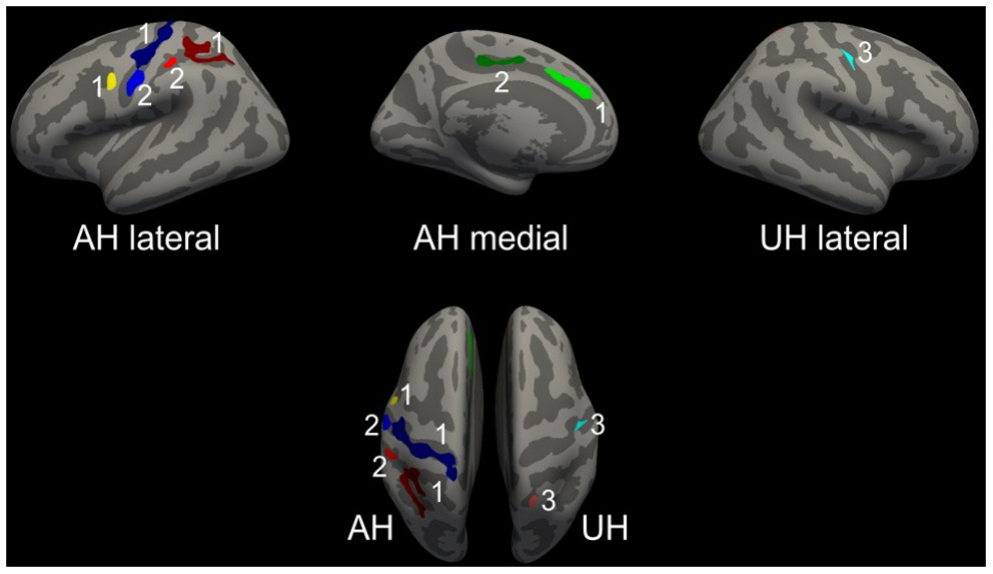
Regions-of-interest for cluster-wise analysis. Selected clusters for ROI analyses. For visualization purposes, the left hemisphere is modeled as the affected hemisphere (AH) and the right hemisphere as the unaffected hemisphere (UH). Central ROIs are displayed in blue tones, the frontal one in yellow, parietal ROIs in red tones and the cingulate ROIs in green. Upper row with lateral and medial views on AH and UH. Lower row with superior view of AH and UH.

Based on a priori hypotheses for functional and structural domains within the motor network with primary and secondary motor areas in frontal, parietal (8, 24) and also cingulate cortices (37–39), significant clusters were extracted for an additional ROI analysis. Figure 3 displays the 9 clusters with significant loss in CT that were chosen: Frontal 1, Central 1–3, Superiorparietal 1–3, Cingulate 1–2. The clusters Central 3 and Superiorparietal 3 were located on the unaffected hemisphere, all others on the ipsilesional hemisphere. For ROI analysis, the large ipsilesional cluster 1 has been divided into clusters Central 1 and Central 2.

### Relationship Between CT, Cortical Excitability, and Clinical Scores

Whole-brain analyses did not detect any significant associations, neither between CT estimates and the TMS data (Slope_max_ and MEP_max_) of AH nor between CT and the clinical scores in the stroke patients. In line, the ROI analysis of the frontal, central, superiorparietal and cingulate motor areas did not uncover any significant relationships with these parameters (Tables 4, 5).

**Table 4:** Patients' linear regression models with the CST's integrity measurements as the response variable.

**Outcome**	**Region**	**Coefficient**	**Conf. Interval**		***P*** **-value**	**Adj. R^2^**
			**Lower**	**Upper**		
Slope_maxAH_	Central 1	0.35	−1.14	1.84	0.61	−0.08
	Central 2	0.30	−0.91	1.52	0.59	−0.08
	Central 3^*^	0.44	−0.5	1.39	0.32	0
	Cingulate 1	0.15	−1.02	1.31	0.79	−0.10
	Cingulate 2	−0.07	−1.23	1.09	0.89	−0.11
	Superiorparietal 1	0.40	−0.95	1.75	0.53	−0.06
	Superiorparietal 2	0.67	−0.34	1.67	0.17	0.09
	Superiorparietal 3^*^	−0.10	−1.28	1.08	0.85	−0.11
	Frontal 1	0.36	−0.48	1.19	0.36	−0.02
MEP_maxAH_	Central 1	0.66	−0.66	1.97	0.29	−0.10
	Central 2	0.57	−0.59	1.73	0.30	−0.11
	Central 3^*^	0.54	−0.36	1.43	0.21	−0.05
	Cingulate 1	0.47	−0.81	1.76	0.43	−0.16
	Cingulate 2	0.19	−0.89	1.28	0.70	−0.22
	Superiorparietal 1	0.72	−0.51	1.96	0.22	−0.06
	Superiorparietal 2	0.78	−0.03	1.59	0.06	0.15
	Superiorparietal 3^*^	0.19	−1.08	1.45	0.75	−0.23
	Frontal 1	0.52	−0.20	1.24	0.14	0.01

* *Central 3 and Superiorparietal 3 are UH clusters. The rest are AH clusters. P-values are uncorrected*.

**Table 5:** Patients' linear regression models with the motor output measurements as the response variable.

**Outcome**	**Region**	**Coefficient**	**Conf. Interval**		***P*** **-value**	**Adj. R^2^**
			**Lower**	**Upper**		
Gripa_AH_	Central 1	−2.77	−35.61	30.06	0.85	0.21
	Central 2	−0.47	−28.68	27.74	0.97	0.21
	Central 3^*^	3.06	−19.44	25.55	0.77	0.21
	Cingulate 1	−1.77	−27.30	23.76	0.88	0.21
	Cingulate 2	−13.49	−37.39	10.41	0.24	0.31
	Superiorparietal 1	−5.06	−36.01	25.88	0.72	0.22
	Superiorparietal 2	3.96	−21.34	29.27	0.73	0.22
	Superiorparietal 3^*^	−4.66	−31.76	22.43	0.71	0.22
	Frontal 1	−2.57	−23.13	17.98	0.79	0.21
Pinch_AH_	Central 1	−0.07	−7.61	7.46	0.98	0.21
	Central 2	0.21	−6.07	6.50	0.94	0.21
	Central 3^*^	0.36	−4.7	5.42	0.88	0.21
	Cingulate 1	−2.18	−7.81	3.46	0.41	0.26
	Cingulate 2	−2.94	−8.45	2.57	0.26	0.30
	Superiorparietal 1	−0.39	−7.48	6.71	0.91	0.21
	Superiorparietal 2	1.68	−3.89	7.25	0.52	0.24
	Superiorparietal 3^*^	−1.18	−7.14	4.79	0.67	0.22
	Frontal 1	−0.13	−4.53	4.28	0.95	0.21
NHP_AH_	Central 1	0.31	−0.12	0.74	0.14	0.13
	Central 2	0.13	−0.27	0.53	0.48	−0.03
	Central 3^*^	0.22	−0.07	0.52	0.12	0.16
	Cingulate 1	0.09	−0.29	0.47	0.60	−0.05
	Cingulate 2	0.17	−0.20	0.54	0.33	0.02
	Superiorparietal 1	0.22	−0.21	0.65	0.28	0.04
	Superiorparietal 2	0.24	−0.05	0.54	0.10	0.19
	Superiorparietal 3^*^	−0.07	−0.46	0.32	0.70	−0.07
	Frontal 1	0.13	−0.14	0.40	0.31	0.03
UEFM	Central 1	4.63	−26.12	35.38	0.75	0.00
	Central 2	−1.10	−27.67	25.48	0.93	−0.01
	Central 3^*^	6.41	−14.58	27.4	0.52	0.03
	Cingulate 1	−7.71	−31.41	15.99	0.49	0.03
	Cingulate 2	−5.09	−29.18	19.00	0.65	0.01
	Superiorparietal 1	0.40	−28.97	29.77	0.98	−0.01
	Superiorparietal 2	3.64	−18.32	25.60	0.72	0.00
	Superiorparietal 3^*^	−12.22	−36.32	11.88	0.29	0.09
	Frontal 1	−1.68	−19.86	16.49	0.84	−0.01

* *Central 3 and Superiorparietal 3 are UH clusters. The rest are AH clusters. P-values are uncorrected*.

## Discussion

The present work shows that chronic stroke patients with isolated subcortical supratentorial stroke lesions exhibit significantly reduced CT in numerous primary and secondary motor and non-motor brain areas, particularly in the ipsilesional hemisphere including areas along the central sulcus, the inferior frontal sulcus, the intraparietal sulcus, and cingulate cortices. However, we could not detect any significant relationship between the extent of these CT reductions and stroke-related changes in motor cortical excitability or clinical scores.

Cortical thinning, especially in brain regions connected to the stroke lesion, has been previously reported by imaging studies. For instance, a longitudinal study comparing stroke patients in the acute phase within three months after stroke found a significant loss in CT in a small area at the superior frontal gyrus at the lateral border of the supplementary motor area (14). However, these CT changes did not correlate with changes in clinical scores. Another work investigated chronic stroke patients and found significant loss in CT in the ipsilesional primary motor cortex. However, this study did not conduct a whole-brain analysis to look for CT alterations in other brain regions (12). Similarly, other studies have limited their CT analyses to pre-specified regions such as the primary motor cortex (5, 17).

In the present cross-sectional whole-brain analysis we show that CT alterations are not limited to the primary motor cortex but are also evident in multiple cortical areas of non-primary motor-related brain regions of the ipsilesional hemisphere. Specifically, we found significant loss in CT in the ventral premotor cortex and in cortices along the intraparietal sulcus. These data are well in line with multiple reports, which have evidenced that the parietofrontal motor network shows relevant functional (23, 40, 41) and structural changes (22, 24) which also relate to recovery after stroke. Moreover, cortices of the cingulate motor areas also showed a significant loss in CT. As cingulate motor areas are structurally and functionally connected with motor, premotor and also somatosensory areas (37, 38, 42), we speculate that network disconnection effects by the stroke lesion are likely to drive these CT reductions as well. In the literature, one study in acute ischemic brainstem strokes also found thinning of the cingulate cortices (18). Previous functional imaging studies have already shown that cingulate motor areas exhibit recovery-dependent increases in brain activation (43, 44), potentially reflecting enhanced processing of somatosensory feedback after stroke (45). Since we have not detected any increases in CT in these areas, but only significant decreases, one might hypothesize that such adaptive processes might be time-specific phenomena and more likely to be relevant in acute or subacute stages than in chronic stages of recovery.

Loss of CT was predominant in, but not limited to, the ipsilesional hemisphere. On the contralesional hemisphere we only detected CT reductions in a few regions in the central sulcus, the cuneal gyrus and calcarine sulcus. This predominance of CT alterations in the ipsilesional hemisphere is in line with a number of previous studies (19, 46). For instance, Cheng et al. estimated an average loss in CT of 0.15 mm after one year in the ipsilesional and 0.13 mm in the contralesional hemisphere in cortices that are structurally connected to the stroke lesions (19).

Some studies have shown that not only loss, but also gain in CT or cortical gray matter volume can be detected after stroke (16, 20, 22). For instance, Liu et al. observed gradual CT increases after basal ganglia stroke in the temporal and frontal lobes (20). The present cohort did not exhibit such increases in CT when compared to the healthy controls. In our study, absolute CT values, and not the relative change of CT over time, were used for group comparison (20). Thus, sensitivity of our cross-sectional approach might be inferior compared to longitudinal statistics to detect subtle gain in CT. Another study found increases of gray matter volume in bilateral supplementary motor areas after right-hemispheric stroke only. These changes also correlated with preserved motor functions (21). A systematic analysis of the influence of the side of the stroke lesion for loss or gain in CT of the affected or unaffected hemispheres would remain a topic for upcoming studies. In line with this idea, precise information regarding subcortical stroke locations, such as striatal vs. non-striatal lesions, might be further influential factors for stroke-related CT alterations in both hemispheres (20). To what extent such information might also influence cross-sectional group comparisons for CT analyses should be addressed by future research.

Earlier reports have been increasingly using multimodal approaches to better understand stroke recovery. For instance, one study investigated the relationship between CT of the primary motor cortex, motor cortical excitability, and clinical measures. While TMS measures could be related to motor functions, CT values could not (5). In the present study, we aimed at addressing this relationship with respect to other cortical brain regions in an exploratory manner. Against our hypothesis, and in accordance with the previously mentioned study, we did not find any significant association, neither between CT and TMS measures of cortical excitability, nor between CT and motor functions after stroke. Concerning the association between CT and clinical scores, there are other studies which could not detect significant correlations (5, 14, 15, 19, 20, 47).

One potential explanation for these negative results is that, although we only included supratentorial subcortical strokes, our patients still exhibited variable lesion locations. Among the affected regions were the corona radiata, internal capsule, basal ganglia, and thalamus. This heterogeneity might still translate into a relevant variability also in the extent of cortical degeneration and atrophy via degeneration of connecting fiber tracts (14, 46). This might complicate the detection of significant relationships spatially converging to a distinct brain region. On the other hand though, even in cohorts with very homogenous stroke locations such as isolated basal ganglia strokes, CT-outcome associations have not been detected (20). This might indicate that CT alone is unlikely to explain a relevant amount of intersubject variability in clinical scores. Hence, other established surrogate markers of the integrity of the motor network, such as fractional anisotropy of the CST, seem to be more informative for residual motor functioning and recovery after stroke (8). Given more recent results for layer-specific CT changes after stroke (13), these negative results could suggest that a higher resolution and the precise CT estimation related to layer V of the primary motor cortex or secondary motor cortices contributing to the CST (48, 49) might be capable to capture such associations between cortical anatomy, electrophysiology, and motor function. For clinical scores, such layer-specific CT estimates have been found to show significant correlations (13).

There are critical limitations to note. First, stroke patients from the initial cohort that had a cortical stroke had to be excluded from the sample. Therefore, only 14 patients could be finally included in the present analyses. On the one hand, this allowed us to interpret CT alterations as secondary degeneration remote from the lesion, because direct lesion effects of the cortical anatomy could be excluded. On the other hand, the reduced sample size influences the power of the statistical analyses. Our results should be verified or falsified in independent datasets. Second, the study was cross-sectional in nature. Longitudinal analyses with repeated sessions of MRI and TMS—throughout the recovery phase—might help to uncover time-dependent associations between cortical thickness and excitability, which undergo changes after stroke. Third, the present cohort included patients with rather mild deficits with a mean NIHSS score of 2 and a mean UEFM score of 56. Thus, whether our findings might be different in stroke patients with more severe deficits, remains a topic for upcoming prospective studies.

## Data Availability Statement

Although there are data sharing restrictions imposed by the ethical review board, data will be made available upon reasonable request, which includes submitting an analysis plan for a secondary project.

## Ethics Statement

The studies involving human participants were reviewed and approved by Ethics Committee of the Chamber of Physicians, Hamburg (No. PV5357). The patients/participants provided their written informed consent to participate in this study.

## Author Contributions

JGP conducted the CT analyses and prepared the manuscript. SG conducted the TMS and imaging experiments and supervised the CT analyses. CC contributed to the statistical analyses. CG contributed to the design of the study. RS contributed to the design of the study, the statistical analyses, and prepared the manuscript. All authors revised the manuscript for important intellectual content.

## Funding

This study was supported by the Werner Otto Stiftung (4/90, RS) and the Deutsche Forschungsgemeinschaft (DFG, German Research Foundation) - 178316478 – C1 (CG). RS and CC were supported by an Else Kröner Exzellenzstipendium from the Else Kröner-Fresenius-Stiftung (Grant Nos. 2020_EKES.16 to RS, 2018_EKES.04 to CC).

## Conflict of Interest

The authors declare that the research was conducted in the absence of any commercial or financial relationships that could be construed as a potential conflict of interest.

## Publisher's Note

All claims expressed in this article are solely those of the authors and do not necessarily represent those of their affiliated organizations, or those of the publisher, the editors and the reviewers. Any product that may be evaluated in this article, or claim that may be made by its manufacturer, is not guaranteed or endorsed by the publisher.
